# Unraveling the Liver–Brain Axis: Resveratrol’s Modulation of Key Enzymes in Stress-Related Anxiety

**DOI:** 10.3390/biomedicines12092063

**Published:** 2024-09-10

**Authors:** Vadim E. Tseilikman, Olga B. Tseilikman, Vadim A. Shevyrin, Oleg N. Yegorov, Alexandr A. Epitashvili, Maxim R. Aristov, Marina N. Karpenko, Ilya A. Lipatov, Anton A. Pashkov, Maxim V. Shamshurin, Irina A. Buksha, Anna K. Shonina, Alexandra Kolesnikova, Vladislav A. Shatilov, Maxim S. Zhukov, Jurica Novak

**Affiliations:** 1 Scientific and Educational Center ‘Biomedical Technologies’, School of Medical Biology, South Ural State University, 454080 Chelyabinsk, Russia; 2 Zelman Institute of Medicine and Psychology, Novosibirsk State University, 630090 Novosibirsk, Russia; 3 Faculty of Fundamental Medicine, Chelyabinsk State University, 454001 Chelyabinsk, Russia; 4 Research, Educational and Innovative Center of Chemical and Pharmaceutical Technologies Chemical Technology Institute, Ural Federal University Named after the First President of Russia B.N. Yeltsin, 620002 Ekaterinburg, Russia; 5 Pavlov Department of Physiology, Institute of Experimental Medicine, 197376 Saint Petersburg, Russia; 6 Federal Neurosurgical Center, 630048 Novosibirsk, Russia; 7 Department of Data Collection and Processing Systems, Novosibirsk State Technical University, 630048 Novosibirsk, Russia; 8 Center for Artificial Intelligence and Cybersecurity, University of Rijeka, 51000 Rijeka, Croatia

**Keywords:** chronic stress, resveratrol, monoamine oxidase, brain, liver, lipid peroxidation, 11*β*-HSD-1, glucocorticoids, anxiety

## Abstract

Stress-related anxiety disorders and anxiety-like behavior in post-traumatic stress disorder (PTSD) are associated with altered neurocircuitry pathways, neurotransmitter systems, and the activities of monoamine and glucocorticoid-metabolizing enzymes. Resveratrol, a natural polyphenol, is recognized for its antioxidant, anti-inflammatory, and antipsychiatric properties. Previous studies suggest that resveratrol reduces anxiety-like behavior in animal PTSD models by downregulating key enzymes such as 11β-hydroxysteroid dehydrogenase type 1 (11β-HSD-1) and monoamine oxidases (MAOs). However, the underlying mechanisms remain unclear. In this study, we explored the efficacy of resveratrol in treating stress-induced anxiety using a chronic predator stress model in rats. Resveratrol was administered intraperitoneally at 100 mg/kg following a 10-day stress exposure, and anxiety behavior was assessed with an elevated plus maze. Our results indicated that stress-related anxiety correlated with increased activities of brain MAO-A, MAO-B, and hepatic 11β-HSD-1, alongside elevated oxidative stress markers in the brain and liver. Resveratrol treatment improved anxiety behavior and decreased enzyme activities, oxidative stress, and hepatic damage. We demonstrate that resveratrol exerts antianxiogenic effects by modulating glucocorticoid and monoamine metabolism in the brain and liver. These findings suggest resveratrol’s potential as a therapeutic agent for anxiety disorders, warranting further clinical investigation.

## 1. Introduction

Anxiety is a primary behavioral response to new stressful events [1]. Its primary function is to help avoid dangerous situations during stressors by triggering the fight-or-flight response [2]. Hence, anxiety as a behavioral response is closely involved in allostasis, which is known as an adaptive process that adjusts homeostasis after acute stress [3]. Allostatic load refers to the physiological “costs” of maintaining allostasis, and allostatic overload denotes an excessive and likely pathological increase in allostatic load [4]. Successful engagement in the fight-or-flight response leads to an allostatic (eustress, in terms of Selye) state. In contrast, failure in the fight-or-flight response leads to allostatic overload (distress, in terms of Selye), which is associated with stress-related behavioral disorders [4]. Depressive, anxiety, and trauma-related disorders share many common symptoms, such as unstable mood, high anxiety, sleep disturbances, and impaired concentration. The high comorbidity of anxiety and depressive disorders has been consistently demonstrated in multiple studies, supporting the argument that depression can be considered a failed anxiety response [5]. Therefore, it is necessary to have cut-off criteria to differentiate between anxiety as an emotional state and anxiety disorders.

In animal post-traumatic stress disorder (PTSD) models, the anxiety index (AI), calculated based on elevated plus maze test (EPM) values, has been successfully used to segregate animals into high- and low-anxiety phenotypes [6]. High-anxiety phenotypes are associated with PTSD vulnerability, whereas low-anxiety phenotypes are associated with PTSD resilience.

The treatment of anxiety disorders primarily relies on the pharmacological management of anxious behavior. Modern pharmacological therapies for anxiety are generally safer and more tolerable than those available 30 years ago. However, despite an enhanced understanding of the pathophysiology of anxiety, the efficacy and duration of these treatments have not significantly improved in most cases. Moreover, despite substantial financial investment in drug development, innovative treatments have yet to reach the market.

A review of the literature on current treatments suggests that evidence-based practice would greatly benefit from more rigorous research into the causes of incomplete treatment responses as well as studies on the comparative efficacy of different drug combinations and sequencing strategies [7]. There are two primary approaches to developing innovative treatments for anxiety: the development of drugs targeting specific neuroreceptors and the pharmacological manipulation of fear-related memory. Unfortunately, neither approach is currently ready for routine clinical use, highlighting the need for novel therapeutic agents to effectively manage anxiety disorders.

It is essential that new drugs possess a multitarget profile, enabling them to modulate various molecular pathways involved in neurotransmitter signaling and the suppression of fear-related memory. Resveratrol, a natural polyphenol and member of the phytoalexin family, meets these criteria well. The wide range of resveratrol’s beneficial biological effects can be attributed to its ability to interact with multiple molecular targets, modulating various intracellular signaling pathways at a micromolar scale.

Recent studies have shown that the efficacy of resveratrol (RES) in correcting PTSD symptoms is comparable to that of selective serotonin reuptake inhibitors, which are known as the first-line treatment for this syndrome [8,9]. RES, a natural polyphenol found in plants such as grapes, has been extensively investigated for its antioxidant, anti-inflammatory, and antipsychiatric properties [10]. Its efficacy in mood correction is attributed to its neuroprotective properties, which involve targeting multiple signaling pathways [11,12].

Sirtuins (SIRT), a family of nicotinamide adenine dinucleotide-dependent deacetylases, mediates many of the effects of RES. The modulation of gene expression in the brain, resulting in neuronal survival, growth, differentiation, and restoration of synaptic activity, occurs through the regulation of histone deacetylase activities in a SIRT1-dependent manner [13]. SIRT1 promotes the activation of AMP-activated protein kinase (AMPK), which is a key regulator of energy homeostasis, metabolism, and cell survival [14,15]. In turn, AMPK mediates mitochondrial biogenesis by upregulating the transcription factor PGC-1α.

SIRT1 also improves synaptic plasticity by activating the expression of the brain-derived neurotrophic factor (BDNF) gene [16,17]. Through targeting the SIRT1/AMPK/PGC-1α pathway, RES enhances mitochondrial function, and by targeting the SIRT1/AMPK/BDNF pathway, it boosts synaptic plasticity [18]. These SIRT1-dependent signaling pathways are crucial in the RES-mediated correction of mitochondrial dysfunction and improvements in synaptic plasticity in experimental PTSD models [19].

Recent studies have shown that RES’s targeting of 11β-hydroxysteroid dehydrogenase type 1 (11β-HSD-1) activity in the liver also contributes to its efficacy in correcting experimental PTSD [20]. 11β-HSD-1 is an NADPH-dependent reductase responsible for the activation of cortisol by reducing cortisone. Data from experimental PTSD in rats demonstrate a correlation between increased 11β-HSD-1 activity in the liver and the severity of behavioral disturbances in PTSD [21]. This association is based on the effect of hepatic 11β-HSD-1 on glucocorticoid levels in the blood. Furthermore, monoamine oxidase A (MAO-A) and monoamine oxidase B (MAO-B) in the brain, known as enzymes that metabolize monoamines, are regulated by glucocorticoids [22]. Thus, the modulation of 11β-HSD-1 activity in the liver by RES impacts the levels of glucocorticoid, which in turn regulates the activities of MAO-A and MAO-B, contributing to the correction of PTSD-related behavioral disturbances.

Stress-related anxiety disorder and anxiety-like behavior in PTSD have both differences and similarities in their underlying mechanisms [23]. PTSD-related anxiety is characterized by long-lasting effects, while stress-related anxiety tends to be a transient effect of stress paradigms. Despite these differences, both types of anxiety behaviors are associated with similar neurocircuitry pathways, neurotransmitter alterations, and the involvement of monoamine and glucocorticoid-metabolizing enzymes [24].

According to DSM-V criteria, stress is always a trigger for PTSD [25]. It has been shown that chronic predator scent stress is a well-validated model of complex PTSD. In this context, RES treatment of PTSD rats allowed the differentiation into two phenotypes: treatment-sensitive rats (TSR) and treatment-resistant rats (TRR) [26]. In TSR rats, *trans*-resveratrol ameliorated anxiety-like behavior and normalized plasma corticosterone concentrations. In contrast, in TRR rats, *trans*-resveratrol aggravated anxiety-like behavior and further decreased plasma corticosterone concentrations. In TSR rats, hepatic 11β-HSD-1 activity was suppressed, which was accompanied by an increase in CYP3A activity. In TRR rats, the activities of both enzymes were suppressed. This suggests that the resistance of PTSD rats to *trans*-resveratrol treatment is associated with abnormalities in the hepatic metabolism of glucocorticoids [26].

Resveratrol administered after the cessation of predator scent exposure improved PTSD-related anxiety. The goal of this study was to test the capability of RES to improve stress-related anxiety and to evaluate the involvement of the 11β-HSD-1/MAO-A pathway in the response of PTSD rats to RES treatment. Anxiety behavior was observed immediately post-exposure, which was accompanied by an increase in the anxiety index.

## 2. Materials and Methods

In this study, we investigated the liver–brain axis in the chronic predator stress (PS) paradigm. To achieve this, we employed a comprehensive methodological approach that included the evaluation of anxiety-like behavior, measurement of corticosterone concentration, assessment of oxidative stress, and determination of enzymatic activities of 11β-HSD-1, MAO-A, and MAO-B. Additionally, liver histology was performed. This approach has been recently and effectively applied in research on post-traumatic stress disorder [27].

### 2.1. Animals

This study was conducted using male Wistar rats housed in individually ventilated, standard cages (3–4 rats per cage). The rats were provided with high-quality rat chow (Beaphar Care Plus Rat Food, Raalte, The Netherlands) and tap water ad libitum. The vivarium maintained a controlled temperature of 22–25 °C, humidity of 55%, and a 12:12 h light–dark cycle with lights on from 7:00 to 19:00.

### 2.2. Chronic Predator Stress Paradigm

In this study, we utilized a chronic PS paradigm. Traditionally, we used cat urine as the source of predator odor for PTSD research. However, this approach did not induce an immediate anxiety state post-cessation of chronic stress exposures. Our previous data suggested that chronic exposure to cat odor led to the desensitization of stressed rats to this stimulus at the end of the PS paradigm, with time-dependent sensitization, a well-known PTSD attribute, becoming evident only two weeks after PS exposure. To induce stress-related anxiety as an urgent behavioral disorder, we replaced cat urine with fox urine.

### 2.3. Fox Urine Collection and Application

The urine of sexually mature males of domesticated silver-black foxes (*Vulpes vulpes*) was used. Urine was collected in the autumn from several males, aliquoted, and stored at −18 °C for no more than one month. The sample was thawed immediately before use. For the stress exposure, 100 µL of urine was applied to a cotton pad, placed in a plastic Petri dish, and covered with a nylon mesh to allow the free distribution of volatile compounds. The Petri dish was placed in the animal’s home cage for 10 min daily for 10 d, starting on day 5 of the experiment.

### 2.4. Resveratrol Administration

*Trans*-resveratrol was purchased from Sigma Aldrich Ltd. (St. Louis, MO, USA). RES injections were performed intraperitoneally daily from days 1 to 10 of the experiment. Solutions were prepared once a week and stored at room temperature. RES, as well as selective serotonin reuptake inhibitors, were dissolved in 99% DMSO to ensure the injection volume corresponded to 1 mL/kg of the animal’s body weight (100 mg/kg).

### 2.5. Experimental Groups

The animals were divided into the following groups:

1. Control: Rats treated with vehicle only for 10 days (*n* = 12).
2. PS: Rats previously exposed to chronic predator stress (*n* = 12).
3. RES + PS: Rats administered an effective dose of resveratrol via intraperitoneal injection one hour before the onset of predatory stress (*n* = 22).

### 2.6. Behavioral Testing

The anxiety levels of all rats were assessed using the EPM test, which was conducted with the standard EPM apparatus TS0502-R3 (OpenScience, Moscow, Russia). The test duration was 10 min. To ensure unbiased results, control and experimental rats were tested together in a blinded manner.

Behavioral recordings and tracking were performed using a SMART video system, and data were analyzed with SMART 3.0 software. Key parameters measured included the following:

- Number of entries into the open and closed arms of the EPM;
- Time spent in the open and closed arms.

From these measurements, the AI was calculated using the following formula [28]:

(1) $$\mathrm{AI}=1-\left\{\frac{\left(\frac{\mathrm{time}\;\mathrm{in}\;\mathrm{open}\;\mathrm{arms}}{\mathrm{total}\;\mathrm{time}\;\mathrm{on}\;\mathrm{maze}}\right)+\left(\frac{\mathrm{number}\;\mathrm{of}\;\mathrm{entries}\;\mathrm{into}\;\mathrm{open}\;\mathrm{arms}}{\mathrm{total}\;\mathrm{number}\;\mathrm{of}\;\mathrm{entries}}\right)}{2}\right\}.$$

Additionally, the number of freezing episodes in the EPM was recorded.

### 2.7. Blood and Tissue Collection and Storage

At the time of necropsy, blood samples and liver tissues were collected from the rats. Blood samples were centrifuged, and the resulting plasma was stored in Eppendorf tubes at −70 °C. Liver tissue was preserved in two ways: 10% buffered formalin for histological analysis and frozen in liquid nitrogen; then, it was stored at −70 °C for subsequent biochemical analyses.

### 2.8. Quantification of RES and RES-O-Glucuronide in Rat Plasma

The quantification of RES and its metabolite, RES-O-glucuronide, in rat plasma was performed using an HPLC system (Agilent 1260 Infinity II, Agilent Technologies, Santa Clara, CA, USA) equipped with a diode array ultraviolet detector. Chromatographic separation was achieved in gradient mode on a Poroshell 120 EC-C_18_ reversed-phase column (3.0 mm × 100 mm × 2.7 µm; Agilent Technologies, 695975-302) with an additional 5 mm guard column. The mobile phase consisted of solvent A (0.1% *v*/*v* acetic acid in water) and solvent B (0.1% *v*/*v* acetic acid in methanol). The proportion of solvent B was linearly increased from 15% to 100% over 14.3 min, which was followed by an isocratic hold at 100% for 1.7 min. The flow rate was maintained at 0.7 mL/min. The column temperature was set at 30 °C, and the injection volume was 5 µL. Analytes were detected at a wavelength of 304 nm.

Standards for constructing the calibration curve were prepared by adding 50 µL of working standard solutions of resveratrol (Sigma-Aldrich, St. Louis, MO, USA, 98%) in methanol to 150 µL aliquots of drug-free plasma. This preparation resulted in plasma standards covering a range of resveratrol concentrations from 0.05 to 20.0 µg/mL. For analysis, 200 µL of plasma or prepared plasma standards were transferred to 2.0 mL centrifuge tubes. To each tube, 50 µL of internal standard solution, containing pterostilbene (Sigma-Aldrich, St. Louis, MO, USA, 97%) at a concentration of 20 µg/mL, was added. The tubes were vortexed for 15 s using a Labtex vortex mixer (Labtex Biotech China Co., Bejing, China). Subsequently, 0.8 mL of acetonitrile was added to each tube, and the mixture was vortexed for an additional 15 s. The tubes were then centrifuged for 15 min at 10,000 rpm using a Thermo ST16R centrifuge (Thermo Scientific, Waltham, MA, USA). The supernatant was transferred to new 2.0 mL tubes and evaporated to dryness at 45 °C under a nitrogen stream using an NDK200 evaporative concentrator (Hangzhou MIU Instruments Co., Hangzhou, China). The dry residues were reconstituted in 0.2 mL of a methanol–water mixture (1:1), transferred to vials, and analyzed.

Plasma RES concentrations were determined using a calibration curve based on the ratio of analyte to internal standard peak areas versus analyte concentration. The concentration of RES glucuronide was estimated using the RES calibration curve, assuming similar signal responses for both analytes. RES and pterostilbene peaks were identified by comparing retention times and ultraviolet spectra (200 to 400 nm) with standard samples.

The identification of resveratrol-O-glucuronide was performed using an Agilent 6545 Q-TOF LC-MS quadrupole time-of-flight mass spectrometer (Agilent Technologies, Santa Clara, CA, USA) equipped with an electrospray ion source in negative ion mode. The chromatographic conditions were adapted to the LC-MS method features, which were similar to those described above. The mass spectrum of resveratrol-O-glucuronide exhibited a deprotonated molecule [M − H]^−^ with an *m/z* of 403.1036 (Δ = 0.42 ppm). In the MS/MS spectrum, obtained under collision-induced dissociation conditions, the loss of the glucuronic acid residue (176 Da) was observed, resulting in the formation of a product ion with an *m/z* of 227.0712 (Δ = 0.74 ppm), corresponding to [M − H]^−^ RES.

### 2.9. Plasma Corticosterone Measurement

Plasma CORT levels were quantified using an enzyme-linked immunosorbent assay (ELISA) kit (Cusabio ELISA Kit, Houston, TX, USA) following the manufacturer’s instructions. The sensitivity of the assay was 0.25 ng/mL with coefficients of variation within and between assays reported as <5%. RES and its metabolite, RES glucuronide, were quantified using high-performance liquid chromatography (HPLC).

### 2.10. Hepatic 11β-HSD-1 Activity

The activity of hepatic 11β-HSD-1 was assessed by measuring the decrease in corticosterone (Sigma Aldrich Ltd., St. Louis, MO, USA) concentration. The assay was conducted using a 0.1 M sodium phosphate buffer (pH 8.5) containing 1.5 mM NADP (Sigma Aldrich Ltd., St. Louis, MO, USA). Incubation was carried out for 60 min at 37 °C. Corticosterone was added to the sample after incubation with a blank sample containing an equivalent volume of solvent for comparison. The changes in fluorescence intensity were measured using a VERSA FLUOR spectrofluorometer (Bio-Rad, Hercules, CA, USA) with excitation at 405 nm and emission at 546 nm [29].

### 2.11. Monoamine Oxidase Activity Measurement

MAO activities and LPO content were measured in isolated brain and liver mitochondria. Mitochondria were obtained from tissue homogenates following the methodology described by Satav and Katyare [30].

Brain MAO activities were assessed in tissue homogenates following the method described by Tipton et al. [31].

For the evaluation of MAO-A activity, brain tissue homogenates were preincubated with 100 µL of 0.5 µM *L*-deprenyl, a selective inhibitor of MAO-B, for 60 min at 37 °C. After preincubation, a specific MAO-A substrate, 5-hydroxytryptamine creatinine sulfate (4 mM), was added to the homogenate.

To inhibit MAO-B activity, 100 µL of 1 µM clorgyline was added to 1 mL of mitochondrial suspension containing MAO in its membrane-bound form and incubated for 60 min at 37 °C. Benzylamine hydrochloride was used as the substrate for MAO-B activity.

The activities of MAO-A and MAO-B were measured spectrophotometrically at 278 nm and expressed as nanomoles of serotonin (MAO-A) or benzylamine hydrochloride (MAO-B) per milligram of protein per minute.

### 2.12. Content of Lipid Peroxidation Products in Liver and Hepatic Mitochondria

The tissue content of LPO products was measured using a spectrophotometric extraction method [32]. This technique differentiates between acyl peroxides and non-esterified fatty acid peroxide intermediates based on their extraction into different solvent phases. Acyl peroxides are extracted from the 2-propanol phase, while non-esterified fatty acid peroxides are extracted from the heptane phase.

The relative content of ketodienes and conjugated trienes was assessed using oxidation indices, specifically the ratio of absorbance at 278 to 220 nm (E278/220). Results are reported as oxidation indices (E278/220), which reflect the relative content of lipid peroxidation products in liver and hepatic mitochondria [32].

### 2.13. Histology

The liver was fixed in 10% neutral formalin, and the samples were processed and embedded in paraffin. Sections were stained with hematoxylin and eosin (H&E) for histological analysis. Tissue morphology was assessed using an Olympus light microscope (Olympus, Tokyo, Japan).

The H&E-stained liver sections were evaluated for inflammation and necrosis by a board-certified pathologist (O.B.T.) who was blinded to the treatment groups. Inflammation was scored based on the presence of macrophage and lymphocyte infiltration. The scoring scale for inflammation was as follows: no inflammation = 1, occasional foci of inflammatory cells = 2, frequently occurring small foci of inflammatory cells = 3, and frequently occurring large foci of inflammatory cells = 4.

Necrosis was assessed based on the percentage of necrotic hepatocytes and the extent of necrotic areas. We used the following necrosis scoring scale: occasional (1%) necrotic hepatocytes = 1, frequent (5–10%) necrotic hepatocytes = 2, small foci of necrosis (clusters of more than 10 necrotic cells) = 3, and extensive areas of necrosis (25% of the lobular unit) = 4 [33].

### 2.14. Statistical Analysis

Data were analyzed using SPSS 24.0 (SPSS Inc., Chicago, IL, USA), STATISTICA 10.0 (StatSoft Inc., Tulsa, OK, USA), and MS Excel 2010 (Microsoft Inc., Redmond, DC, USA) software. Quantitative data are presented as mean ± standard deviation (SD). The Kruskal–Wallis test was used for comparisons among groups, which was followed by Dunn’s post hoc tests for pairwise comparisons. Spearman correlations were calculated to assess the relationships between variables.

## 3. Results

### 3.1. Timeline of PS Exposures, RES Treatment, and Blood RES Concentration Evaluation

**Figure 1:**
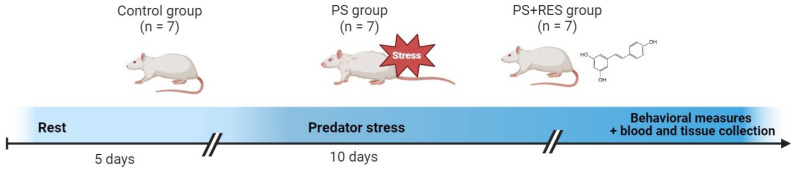
Timeline of predator stress exposures.

To assess the impact of RES treatment on anxiety-like behavior and its metabolic correlates—such as 11β-HSD-1 in the liver, and cerebral MAO-A, MAO-B, and lipid peroxidation (LPO) contents in the brain—as well as on liver injury caused by chronic stress, we initiated a 10-day exposure of rats to predator stress (PS) (Figure 1).

**Figure 2:**
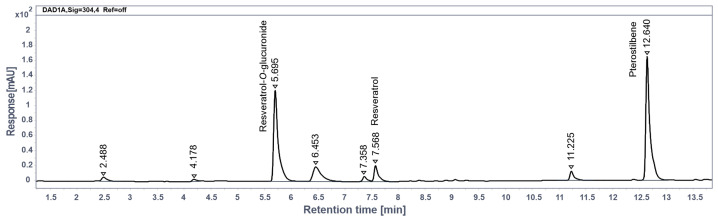
RES and RES glucuronide concentration in blood by HPLC data.

The PS-subjected rats were divided into two groups—treated with daily intraperitoneal injections of RES at a dose of 100 mg/kg (“PS + RES” group) and treated with vehicle only (“PS” group). Rats in the control group also received vehicle injections. In the plasma of the “PS + RES” rats, concentrations of RES and its main metabolite, RES glucuronide, were evaluated (Figure 2). We tested for correlations between RES and RES glucuronide concentrations and various behavioral, metabolic, and histological parameters to understand the impact of RES treatment on anxiety-like behavior and its metabolic correlates.

### 3.2. Evaluation of Behavioral Effects of RES Treatment on PS Rats

The data presented in Table 1 suggest an anti-anxiety effect of RES treatment in PS rats. One-way ANOVA revealed significant changes in the time spent in the open arms of the maze (F(2,19) = 6.39, *p* = 0.007) and the time spent in the closed arms (F(2,19) = 5.82, *p* = 0.01). Additionally, there were significant changes in the number of entries into the closed arms (F(2,19) = 5.94, *p* = 0.034) and the AI value (F(2,19) = 9.4, *p* = 0.0014). The freezing behavior also showed significant changes (F(2,19) = 7.77, *p* = 0.0034) in the EPM test. Notably, PS rats spent twice as much time in the closed arms of the maze compared to control rats (*p* < 0.05), indicating increased anxiety-like behavior.

Meanwhile, PS rats spent 57% less time in the open arms than the control rats (*p* < 0.05). The number of entries into the closed arms in PS rats was 2.5 times higher than in the control group (*p* < 0.001). The AI of the PS rats was twice as high as that of the control rats (*p* < 0.005). Moreover, PS rats exhibited a 6.4-fold increase in the number of freezing acts in the maze (*p* < 0.005). RES treatment reversed the PS-induced shifts in the EPM test. In the “PS + RES” group, post hoc analyses revealed a 41% decrease in the time spent in the closed arms (*p* < 0.05) with a simultaneous 255% increase (*p* < 0.005) in the time spent in the open arms (*p* < 0.05) compared to the “PS” group. Additionally, in the “PS + RES” rats, the AI value was reduced by 43% (*p* < 0.05) compared to the control group.

**Figure 3:**
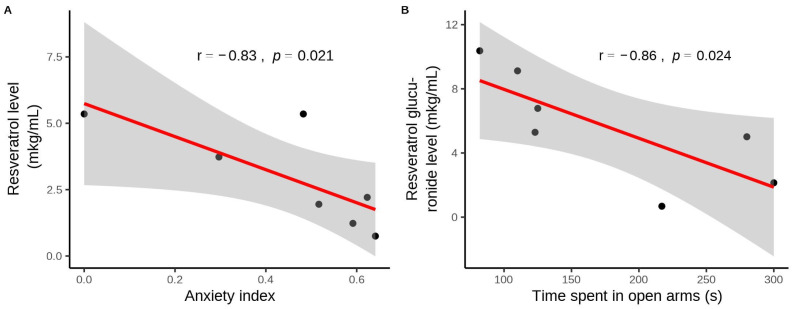
(**A**) Correlation plot showing the relationship between resveratrol levels and the anxiety index in PS rats. (**B**) Association between resveratrol glucuronide levels and time spent in the open arms of the elevated plus maze. The gray zone surrounding the red line represents the 95% confidence interval.

In the treated PS rats, there was a significant negative correlation between the AI and plasma RES concentration (r = −0.83, *p* < 0.05) (Figure 3A). Similarly, a negative correlation was observed between the concentration of plasma RES glucuronide, the main metabolite of RES in tissues, and the time spent in the open arms of the EPM (r = −0.86). RES treatment resulted in the complete disappearance of freezing behavior in PS rats (*p* < 0.005). One-way ANOVA analysis also indicated significant differences in freezing behavior due to the PS paradigm and RES treatment (F(2,19) = 7.78, *p* = 0.0038). In PS rats, the number of freezing acts increased eightfold (*p* < 0.005), whereas RES treatment completely eliminated freezing behavior in the EPM test (*p* < 0.001).

### 3.3. Effects of RES Treatment on Plasma CORT Concentration in PS Rats

**Figure 4:**
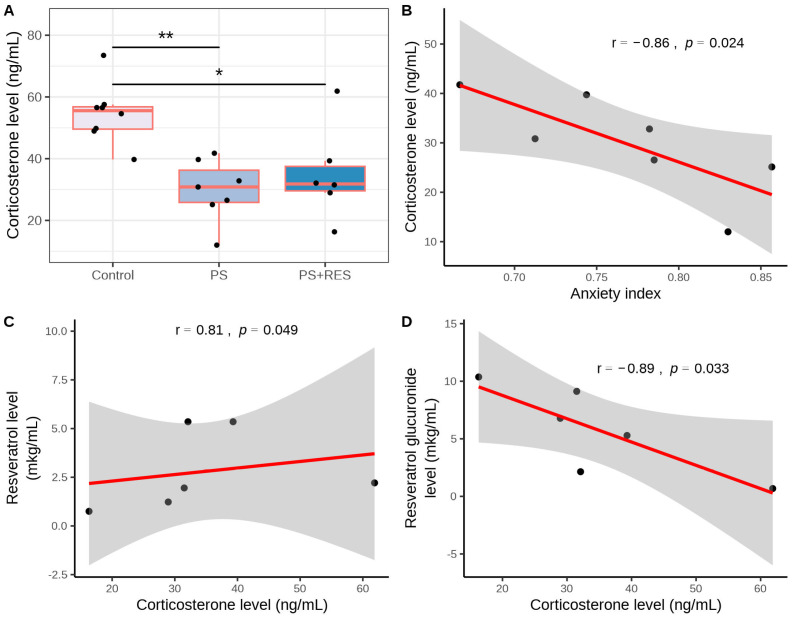
Impact of RES treatment on plasma CORT concentration in PS rats and correlations among CORT concentration, anxiety index, and plasma RES and RES glucuronide concentrations. (**A**) Plasma CORT concentration in RES-treated versus untreated PS rats. (**B**) Correlation plot illustrating the relationship between CORT concentration and the anxiety index in PS rats. (**C**) Correlation plot depicting the relationship between CORT concentration and RES concentration in PS rats. (**D**) Correlation plot showing the relationship between CORT concentration and RES glucuronide concentration in PS rats. * *p* < 0.04; ** *p* < 0.007. The gray zone surrounding the red line represents the 95% confidence interval.

Figure 4 illustrates the impact of the PS paradigm on corticosterone (CORT) concentration ($\chi^{2}$ = 10.48, *p* = 0.005). Significant differences were also observed in hepatic 11β-HSD-1 activity ($\chi^{2}$ = 11.89, *p* = 0.0026) and 11β-HSD-1 protein concentration ($\chi^{2}$ = 8.97, *p* = 0.011).

Post hoc analysis revealed that the plasma CORT concentration in PS rats was 55% lower (*p* < 0.005) than in the control group (Figure 4A). RES treatment did not reverse the reduction in plasma CORT concentration in PS rats (Figure 4A). In the “PS + RES” group, plasma CORT concentration was 35% lower than in the control group (*p* < 0.01). However, no significant difference was observed between untreated and RES-treated PS rats (*p* > 0.05). A negative correlation was found between AI and plasma CORT concentration in PS rats (r = −0.86, *p* < 0.05) (Figure 4B). Additionally, a positive correlation was indicated between plasma CORT and plasma RES concentrations in RES-treated rats (r = 0.81, *p* < 0.05) (Figure 4C). Simultaneously, a negative correlation was revealed between plasma concentrations of RES glucuronide and plasma CORT concentration (r = −0.73, *p* < 0.05) (Figure 4D).

### 3.4. Effects of RES Treatment on Hepatic 11β-HSD-1 Activity and Expression in PS Rats

**Figure 5:**
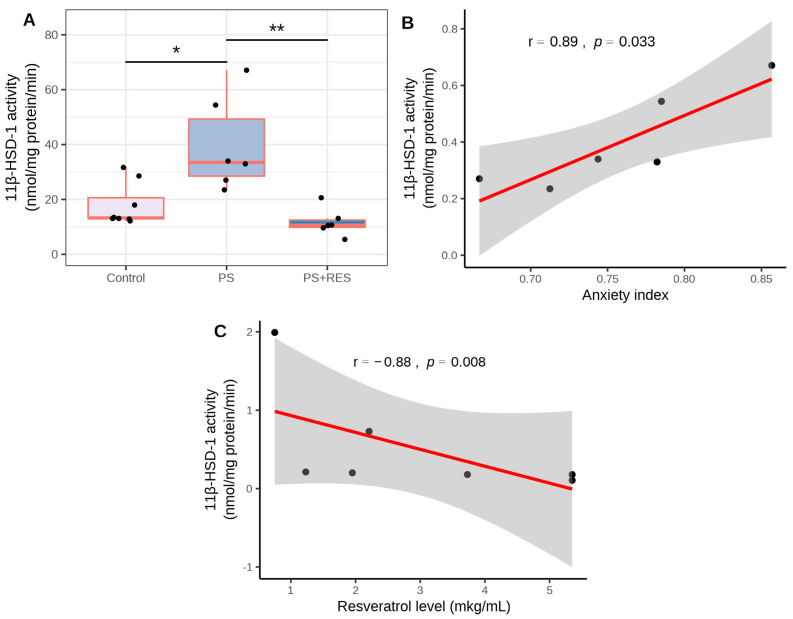
Impact of RES treatment on hepatic 11β-HSD-1 activity in PS rats and correlations among enzymatic activity, anxiety index, and plasma RES concentration. (**A**) Hepatic 11β-HSD-1 activity in RES-treated versus untreated PS rats. (**B**) Correlation plot showing the relationship between 11β-HSD-1 activity and the anxiety index in PS rats. (**C**) Correlation plot depicting the relationship between 11β-HSD-1 activity and RES concentration in PS rats. * *p* < 0.05; ** *p* < 0.002. The gray zone surrounding the red line represents the 95% confidence interval.

The data presented in Figure 5 demonstrate the effect of RES treatment on hepatic 11β-HSD-1 activity ($\chi^{2}$ = 11.89, *p* = 0.0026) and 11β-HSD-1 protein concentration ($\chi^{2}$ = 8.97, *p* = 0.011) in PS rats. In PS rats, hepatic 11β-HSD-1 activity was 215% higher (*p* < 0.005) than in control rats (Figure 5A). In the “PS + RES” group, hepatic 11β-HSD-1 activity was 71% lower (*p* < 0.0005) than in the “PS” group (Figure 5A). Positive correlations were observed between hepatic 11β-HSD-1 activity and the AI (r = 0.88, *p* < 0.05) and between hepatic 11β-HSD-1 activity and time spent in the closed arms of the EPM (r = 0.82, *p* < 0.05) in untreated PS rats (Figure 5B,C).

**Figure 6:**
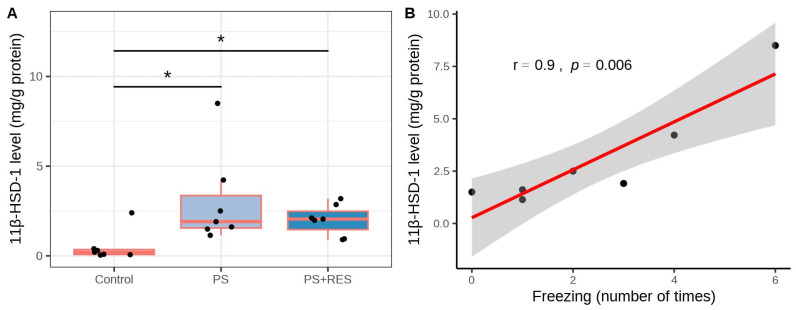
Impact of RES treatment on hepatic 11β-HSD-1 protein levels in PS rats. (**A**) Hepatic 11β-HSD-1 protein concentration in RES-treated versus untreated PS rats. (**B**) Correlation plot showing the relationship between 11β-HSD-1 protein concentration and freezing behavior in PS rats. * *p* < 0.03. The gray zone surrounding the red line represents the 95% confidence interval.

Additionally, a positive correlation was identified between 11β-HSD-1 protein concentration and freezing behavior in PS rats (r = 0.81, *p* < 0.05) (Figure 6).

### 3.5. Effects of RES Treatment on MAO-A and MAO-B Activities in PS Rats

**Figure 7:**
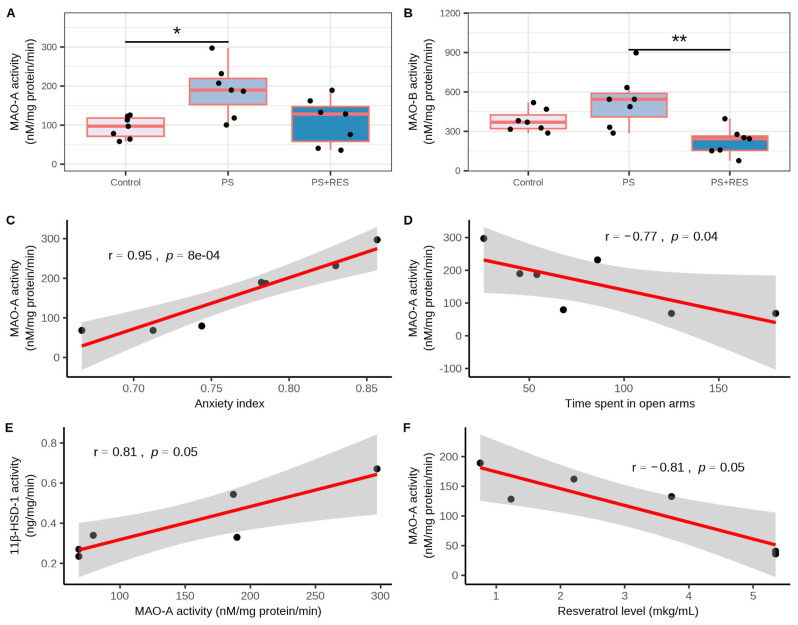
Impact of RES treatment on brain MAO-A/MAO-B activities. (**A**) Brain MAO-A activity in RES-treated versus untreated PS rats. (**B**) Brain MAO-B activity in RES-treated versus untreated PS rats. (**C**) Correlation plot showing the relationship between MAO-A activity and the anxiety index. (**D**) Correlation plot showing the relationship between MAO-A activity and time spent in open arms. (**E**) Correlation plot showing the relationship between MAO-A activity and hepatic 11β-HSD-1 activity. (**F**) Correlation plot showing the relationship between MAO-A activity and RES concentration in plasma. * *p* < 0.05; ** *p* < 0.01. The gray zone surrounding the red line represents the 95% confidence interval.

In treated PS rats, a negative correlation was observed between plasma RES concentration and hepatic 11β-HSD-1 activity (r = −0.88, *p* < 0.05) (Figure 5D). In PS rats, the concentration of 11β-HSD-1 protein in the liver (Figure 6A) was six times greater than in control rats (*p* < 0.01). RES treatment failed to reverse the overexpression of 11β-HSD-1 (*p* > 0.05). Figure 7 demonstrates the ability of RES treatment to downregulate changes in brain MAO-A and MAO-B activities in PS rats. Significant differences were observed in MAO-A ($\chi^{2}$ = 7.24, *p* = 0.027) and MAO-B ($\chi^{2}$ = 11.28, *p* = 0.0035) activities in the brain. In untreated PS rats, brain MAO-A activity (Figure 7A) doubled (*p* < 0.005), while brain MAO-B activity (Figure 7B) increased by 138% (*p* < 0.005) compared to control rats. RES treatment reduced MAO-A activity by 45% (*p* < 0.01) and decreased MAO-B activity by 42% (*p* < 0.01) compared to PS rats. In PS rats, positive correlations were observed for brain MAO-A activity with the AI (r = 0.95, *p* < 0.05), time spent in closed arms (r = 0.75, *p* < 0.05), and hepatic 11β-HSD-1 activity (r = 0.75, *p* < 0.05) (Figure 7C–E). Additionally, a negative correlation was found between brain MAO-A activity and plasma RES concentration (r = −0.81, *p* < 0.05) (Figure 7F).

### 3.6. Effects of RES Treatment on Brain LPO Levels in PS Rats

**Figure 8:**
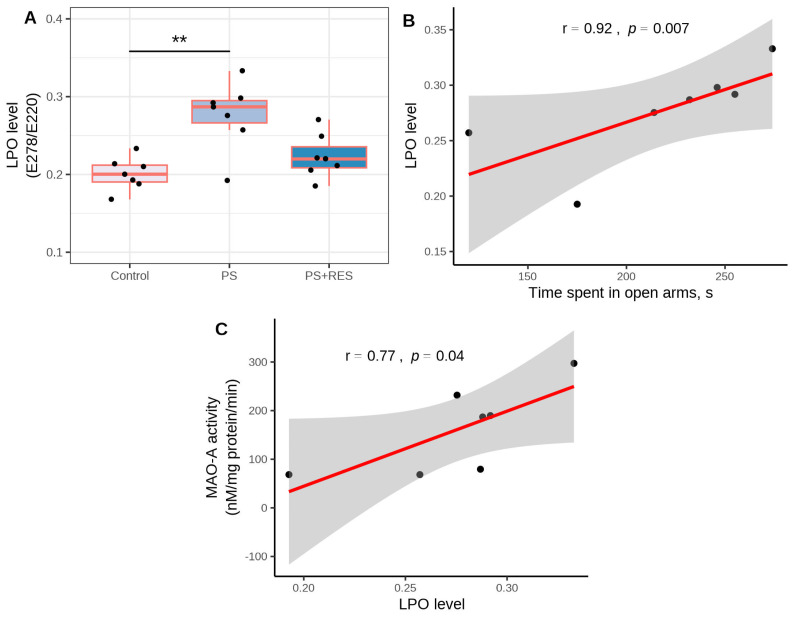
Impact of RES treatment on brain LPO levels. (**A**) Brain LPO levels in RES-treated versus untreated PS rats. (**B**) Correlation plot showing the relationship between LPO levels and time spent in the open arms. (**C**) Correlation plot showing the relationship between LPO levels and brain MAO-A activity. ** *p* < 0.01. The gray zone surrounding the red line represents the 95% confidence interval.

Figure 8 illustrates the efficiency of RES treatment in relation to alterations in LPO levels in the brain ($\chi^{2}$ = 9.04, *p* = 0.011) and liver ($\chi^{2}$ = 9.35, *p* = 0.0023) of PS rats. In untreated PS rats (Figure 8A), brain LPO levels were 142% higher than in control rats (*p* < 0.0005). In the “PS + RES” group, LPO levels were 11% lower than in the “PS” group (*p* < 0.01). A positive correlation between brain LPO levels and time spent in the closed arms (r = 0.96, *p* < 0.05) was indicated in PS rats. Notably, a positive correlation between MAO-A activity and LPO levels (r = 0.78, *p* < 0.05) in the brain of PS rats was also revealed.

### 3.7. Effects of RES Treatment on Liver Histology and LPO Levels in PS Rats

In the liver, significant differences in LPO levels between the control and all experimental groups were indicated (F(2,15) = 9.35, *p* = 0.0023). In the “PS” group (Figure 8B), the level of hepatic LPO was 325% higher than in control groups (*p* < 0.001). RES treatment significantly reduced oxidative stress in the liver. In the “PS + RES” group, LPO levels were 43% lower than in PS rats (*p* < 0.01).

**Figure 9:**
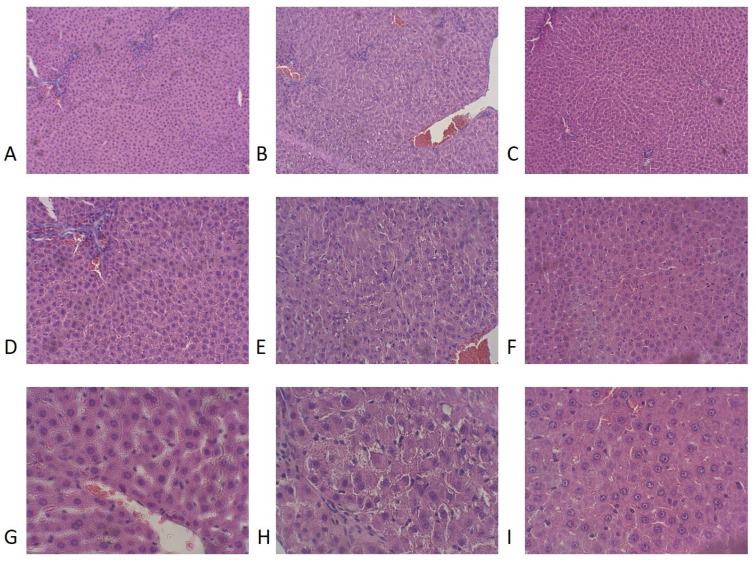
Liver condition in animals of the control group (**A**–**C**), PS group (**D**–**F**), and PS + RES group (**G**–**I**) rats. (**A**) Rat liver from the control group. Stained with hematoxylin and eosin. (E*100) No significant morphological changes observed. Note the slight uneven fullness. 1—portal tract; 2—signs of fullness of portal vessels; 3—uneven fullness of hepatic sinusoids. (**B**) Rat liver from the control group. Stained with hematoxylin and eosin. (E*200) No significant morphological changes observed. Note the slight uneven fullness. 1—portal tract; 2—signs of fullness of portal vessels; 3—uneven fullness of hepatic sinusoids. (**C**) Rat liver from the control group. Stained with hematoxylin and eosin. (E*400) No significant morphological changes observed. Note the slight uneven fullness. 1—hepatocytes; 2—shaped elements of blood. (**D**) Rat liver from a group of animals subjected to stress. Stained with hematoxylin and eosin. (E*100) Significant liver damage. 1—pronounced uneven fullness; 2—sites of necrobiotic and necrotic changes in hepatocytes; 3—foci of perifocal lymphomacrophageal infiltration. (**E**) Rat liver from a group of animals subjected to stress. Stained with hematoxylin and eosin. (E*200) Significant liver damage. 1—pronounced uneven fullness; 2—sites of necrobiotic and necrotic changes in hepatocytes; 3—lymphomacrophageal elements. (**F**) Rat liver from a group of animals subjected to stress. Stained with hematoxylin and eosin. (E*400) Significant liver damage. 1—focus of hepatocyte death; 2—perifocal infiltration. (**G**) Rat liver from a group of stressed animals treated with resveratrol. Stained with hematoxylin and eosin. (E*100) Moderate liver damage with intensification of hepatocyte proliferation. 1—section of fibrin-like masses; 2—small areas of necrobiotic and necrotic phenomena; 3—diffuse minor lymphomacrophageal infiltration with an admixture of single eosinophils and neutrophils; 4—hepatocytes with large nuclei. (**H**) Rat liver from a group of stressed animals treated with resveratrol. Stained with hematoxylin and eosin. (E*200) Moderate liver damage with intensification of hepatocyte proliferation. 1—small area of necrobiotic and necrotic phenomena; 2—focal diffuse infiltration; 3—hepatocytes with large nuclei. (**I**) Rat liver from a group of stressed animals treated with resveratrol. Stained with hematoxylin and eosin. (E*400) Moderate liver damage with intensification of hepatocyte proliferation. 1—hepatocytes with large nuclei showing chromatin condensation phenomena and noticeable nucleoli.

The data presented in Table 2 and Figure 9 show histological alterations in the liver of PS rats treated with RES. One-way ANOVA analysis indicated significant differences (Table 2) in the number of macrophage cells (F(2,19) = 6.79, *p* = 0.025), lymphoid cells (F(2,19) = 9.48, *p* = 0.001), and necrotic foci (F(2,19) = 11.11, *p* = 0.0025).

**Figure 10:**
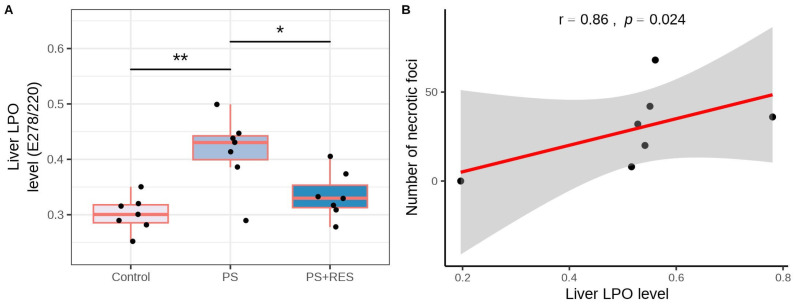
Impact of RES treatment on hepatic LPO levels. (**A**) Comparison of hepatic LPO levels between RES-treated and untreated PS rats. (**B**) Correlation plot illustrating the relationship between LPO levels and the number of necrotic foci. * *p* < 0.05; ** *p* < 0.005. The shaded gray area around the red line indicates the 95% confidence interval.

PS rats had elevated LPO levels compared to control groups, which were reversed by RES treatment (Figure 10A). In the liver of PS rats, mononuclear cell infiltration was evident, which was manifested by a tenfold increase in the number of lymphocytes (*p* < 0.0005) and a fourfold increase in the number of monocytes (*p* < 0.0005) compared to the control group. Control rat livers showed no evidence of necrotic foci in hepatocytes, whereas stressed rats exhibited extensive areas of necrosis, encompassing more than 25% of the area unit (*p* < 0.001). A positive correlation (Figure 10B) was observed between necrotic foci in rat hepatocytes and liver LPO levels (r = 0.86, *p* < 0.024). RES treatment significantly reduced necrotic foci in the hepatocytes of PS rats (Table 2). In the “PS+RES” group, the number of necrotic foci decreased by 95% (*p* < 0.001) compared to PS rats. Simultaneously, in RES-treated rats, the number of macrophage cells and lymphocyte cells decreased by 51% compared to untreated PS rats (*p* < 0.05).

In summary, the obtained results highlight the significance of the liver–brain axis in the pathogenesis of stress-related anxiety disorders. The study underscores the unique potential of RES in treating anxiety disorders by targeting liver 11β-HSD-1 and brain MAO-A. Additionally, RES treatment was shown to downregulate inflammatory processes and oxidative stress, contributing to the correction of liver damage.

## 4. Discussion

Our research utilized a comprehensive approach to unravel the mechanisms underlying stress-related anxiety by examining the interplay between hepatic 11β-HSD-1 and brain MAO-A/B enzymes. These enzymes play a critical role in the downregulation of glucocorticoids and monoamines. Specifically, 11β-HSD-1 modulates glucocorticoid levels, which, depending on various conditions, can either enhance or reduce the activity of brain MAOs, influencing neurotransmitter concentrations and overall anxiety levels. RES, due to its ability to target both 11β-HSD-1 and MAOs, may help to limit anxiety behaviors.

This study presents data suggesting the protective effects of RES treatment on stress-related anxiety behavior in a chronic PS paradigm. Correlation analysis revealed links between the concentrations of RES and its metabolite RES glucuronide with the evaluated behavioral, metabolic, and histological parameters. In PS rats, stress-related anxiety was assessed using the EPM test and was primarily manifested by a significant increase in the AI value. The elevated AI was due to increased time spent in the open arms, decreased time spent in the closed arms, and increased entries into the closed arms of the EPM. Additionally, an increase in freezing behavior was observed in PS rats. Freezing in the EPM test may reflect a fear response [34]. The relationship between freezing and anxiety can be understood in two ways: antagonism or synergism [34]. We hypothesize that antagonism between AI and freezing, with a predominance of anxiety signs, characterizes the propensity to act according to the “fight or flight” response, thus considering anxiety as a eustress attribute. Conversely, synergism between freezing and AI indicates helplessness in executing the “fight or flight” response, suggesting anxiety as a distress attribute. Given the simultaneous increase in anxiety and freezing, it is reasonable to assume anxiety disorders in PS rats.

In PS rats, anxiety disorders are associated with increased activities of brain MAO-A and MAO-B. There is substantial evidence suggesting that MAO enzymes play a crucial role in anxiety behavior, although findings in this area often contradict each other. For instance, MAO-A and MAO-B knockout mice exhibit increased stress reactivity [35]. Chen and colleagues reported a significant increase in anxiety-like behavior in MAO-A/B double knockout mice [36]. These findings align with the role of endogenous MAO inhibitors, known as tribulins, in inducing anxiety behavior [37]. Moreover, it has been observed that low-anxiety phenotype rats are characterized by increased brain MAO enzyme activity [38].

Recent studies have reported decreased brain MAO-A activities in the PS paradigm, which is an established animal model of PTSD. Conversely, in the single prolonged stress model, another animal PTSD model, increased brain MAO-A/B activities have been documented [39]. Glucocorticoid signaling in different brain areas can increase MAO-A mRNA levels via the SIRT1/NHLH2/MAO-A pathway, promoting anxiety-like behavior [40]. SIRT1 in the brain directly influences mood and behavior by deacetylating the NHLH2 transcription factor, which activates MAO-A transcription [41].

Thus, anxiety-like behavior may be synchronized with either decreased or increased brain MAO expression/activity, depending on the context. Alterations in MAO-A/MAO-B activities affect the balance between brain monoamines, which in turn provoke stress-related anxiety. Increased MAO-A/MAO-B activities can exacerbate anxiety behavior by inducing oxidative stress [42]. MAO enzymes, residing in the mitochondrial membrane, generate hydrogen peroxide as a by-product of monoamine oxidative deamination [43]. Consequently, MAO activation is associated with increased LPO, exacerbating mitochondrial oxidative stress and promoting mitochondrial dysfunction [44].

Mitochondria are critical regulators of neural structure and function [45], and evidence suggests that mitochondrial dysfunction in high anxiety is accompanied by elevated levels of LPO products [46]. In this study, the positive correlations between AI, MAO-A, MAO-B activities, and mitochondrial LPO levels in PS rats support this perspective.

The current PS paradigm is characterized by low plasma CORT concentrations, which complements earlier data from our lab indicating a reduction in glucocorticoids. However, previous studies focused on anxiety-like behavior as a sign of PTSD, performing the EPM test two weeks after the cessation of the PS paradigm [47]. In contrast, this study evaluates anxiety levels and plasma CORT concentrations as behavioral and endocrinological signs of chronic stress, rather than PTSD, since all experimental procedures were conducted immediately post-PS paradigm.

Low CORT concentration is associated with increased liver 11β-HSD-1 activity/expression [48]. Recent findings indicate a molecular pathway linking hepatic 11β-HSD-1 to brain MAO-A with plasma CORT involvement in anxiety development. In this pathway, 11β-HSD-1 acts as a hepatic trigger of stress-related anxiety behavior. This is supported by consistent positive correlations between AI and liver 11β-HSD-1 activity with values ranging from 0.8 to 0.9 in this and previous studies. The positive correlations between 11β-HSD-1 activity/protein concentration and AI suggest that hepatic 11β-HSD-1 plays a role in the pathogenesis of stress-related anxiety disorders.

Earlier studies showed that unstressed high-anxiety rats have higher levels of 11β-HSD-1 activity and 11β-dehydrocorticosterone (a 11β-HSD-1 metabolite) compared to unstressed low-anxiety rats [47]. Additionally, it has been demonstrated that increased hepatic 11β-HSD-1 activity causes low plasma CORT levels in the PS paradigm, which is an animal model of PTSD [27]. Therefore, stress-related anxiety behavior and PTSD-like symptoms share common underpinnings, which are manifested in increased hepatic 11β-HSD-1 activity leading to reduced plasma CORT concentration.

Low glucocorticoid levels are associated with high levels of proinflammatory cytokines, potentially leading to inflammation. In the PS paradigm, low CORT levels increase the risk of proinflammatory complications in internal organs. In the liver, stress induces necrotic foci with perivascular and lobular infiltration of mononuclear cells along with increased free radical oxidation. The negative correlation between plasma CORT concentration and mononuclear cells supports the involvement of low glucocorticoid levels in the deleterious effects of chronic PS paradigm.

The main advantage of this study is the opportunity to correlate the effects of RES treatment on the behavioral, endocrine, metabolic, and histological parameters with RES and RES glucuronide concentrations in the blood. RES treatment significantly improved most of the observed behavioral complications, preventing the increase in AI value and eliminating freezing behavior in PS rats. The negative correlation between AI value and RES concentration indicates a link between the antianxiogenic effect of RES treatment and the pharmacokinetics of this stilbenoid. Our data align well with other research demonstrating the effectiveness of RES treatment on stress-related anxiety behavior [49].

It is well known that the majority of RES’s protective effects are mediated by sirtuin (SIRT). Despite the presence of multiple molecular targets for RES, SIRT proteins stand out due to the crucial role of SIRT1 in maintaining genome integrity, as evidenced by studies on SIRT1 knockout mice [50]. Notably, SIRT1 has a dual influence on stress-related anxiety behavior. The main molecular pathways supporting the anti-anxiety effects of RES treatment include the SIRT1/AMPK/CREB/BDNF pathway, which helps restore neurotransmitter balance, improve synaptic plasticity, increase mitochondrial biogenesis, and enhance glucose uptake by neuronal cells [51]. Yuan et al. [9] reported that RES increased cAMP response element-binding protein (CREB) and BDNF levels, which were decreased in mice subjected to single-prolonged stress [50]. Our previous data suggested that RES promotes an increase in BDNF gene expression in the hippocampus of PS rats [34]. It is likely that the antianxiogenic effect of RES treatment is associated with enhancing the SIRT1/AMPK/CREB/BDNF pathway in PS rats [52].

Additionally, RES impacts the SIRT1/NHLH2/MAO-A pathway, which is known to enhance anxiety-like behavior. RES treatment suppressed the elevation of brain MAO-A activity in PS rats [53].

In silico data suggest that RES can directly bind to certain sites of MAO-A and inhibit its catalytic activity [54]. This aligns well with in vivo data indicating that RES inhibits noradrenaline and 5-hydroxytryptamine uptake and monoamine oxidase activity [55]. In the current study, the negative correlation between plasma RES concentration and brain MAO-A activity reflects the direct influence of RES on the metabolism of monoamines in the brain. Another attribute of RES treatment is the reduction of hepatic 11β-HSD-1, which was evidenced by the negative correlation between plasma RES concentration and the activity of this enzyme in the liver. Recent studies have shown that RES can improve anxiety-like behavior in animal PTSD models, suggesting that hepatic 11β-HSD-1 and brain MAO-A are common targets for RES in both contexts [27].

The inhibitory effects of RES on hepatic 11β-HSD-1 are manifested in its activity but not in its expression. Despite the effective correction of 11β-HSD-1 activity, RES treatment does not prevent the decrease of plasma CORT concentration in PS rats. Based on the negative correlation between CORT and RES glucuronide concentrations in plasma, it is assumed that the rapid metabolism of RES by microsomal enzymes limits its effectiveness in preventing glucocorticoid reduction in PS rats.

It is important to note that glucocorticoid can be metabolized in the liver via the CYP3A pathway apart from 11β-HSD-1. RES also downregulates CYP3A activity, but the rapid transformation of RES to RES glucuronide may prevent it from effectively suppressing the CYP3A-dependent metabolism of glucocorticoid [56]. Notably, the predominance of the CYP3A-dependent pathway over the 11β-HSD-1 pathway in glucocorticoid metabolism is associated with low-anxiety phenotypes in rats [47].

Although RES treatment does not counteract the reduction of CORT concentration in PS rats, liver damage was significantly diminished in the “PS + RES” group. Notably, a negative correlation was revealed between plasma RES concentration and necrotic foci, which was likely linked to the antioxidant effects of RES treatment on the liver. In the “PS+RES” group, reduced levels of LPO in the liver were observed, supporting this assumption through the positive correlation between necrotic foci and LPO levels.

Previous studies have indicated liver damage in restraint stress paradigms, which were also accompanied by mononuclear cell infiltration. It has been reported that the IL-1 receptor antagonist Anakinra, at a dose of 2 µg/kg, can prevent the infiltration of mononuclear cells and reduce free radical oxidation and necrosis of lesions in stressed conditions [57]. Blocking IL-1 receptors with an antagonist significantly rescues stress-induced liver injury. Similarly, the hepatoprotective effects of RES in PS rats might also be due to the reduction of proinflammatory cytokine signaling.

This hypothesis is supported by data suggesting that targeting the ROS/NF-κB signaling pathway with RES may lead to a significant improvement of hepatic injury [58]. The hepatoprotective effect of RES might also play a role in correcting anxiety-like behavior in stressed rats. This assumption is based on the recently unveiled link between hepatic damage and heightened anxiety in PTSD rats [27].

**Figure 11:**
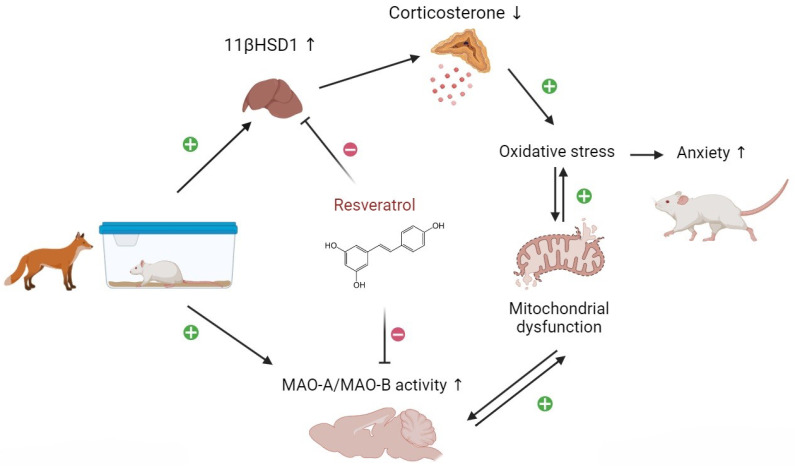
Possible mechanism of the anxiolytic effect of RES treatment in PS rats, illustrated by upregulation (upward arrows) or downregulation (downward arrows) of relevant molecular targets.

Figure 11 illustrates the proposed mechanism of RES’s dual effect on anxiety disorders, which targets the liver–brain axis, starting with hepatic 11β-HSD-1 and culminating in brain MAO-A. The protective effects of RES are linked to its direct binding with 11β-HSD-1 and MAO proteins as well as its ability to upregulate the SIRT1/AMPK/CREB/BDNF pathway.

The interactions between 11β-HSD-1 activity and the AMPK/SIRT1 signaling pathways are not well understood. However, it has been shown that a selective 11β-HSD-1 inhibitor can mimic the RES-mediated promotion of the AMPK/SIRT1 signaling pathways [59]. The antioxidant properties of RES may contribute to correcting mitochondrial dysfunction. In this study, RES prevented the overproduction of LPO in brain and liver mitochondria. Notably, RES acts as a free radical scavenger by invading mitochondria, further enhancing its antioxidant effects [60].

Additionally, RES enhances mitochondrial function via activation of the SIRT1/AMPK/PGC-1α pathway [61]. Specifically, RES targets SIRT1, which subsequently upregulates AMPK through liver kinase B1 serine threonine kinase STK11 (also called LKB1). AMPK promotes the protective effects of PGC-1α on mitochondria [61]. The protective effects of RES treatment on mitochondria may be reflected in the activities of MAO-A and MAO-B, which are mitochondrial enzymes. Mitochondrial dysfunction may affect MAO-A/MAO-B activities, and altered MAO enzyme activities can, in turn, exacerbate mitochondrial dysfunction. Thus, the simultaneous normalization of both enzymes’ activities in PS rats might be due to improved mitochondrial function following RES treatment.

Although this article primarily focuses on the effects of resveratrol on liver 11β-HSD-1 and brain MAO-A/B, it does not exclude the involvement of other potential biological mechanisms. Anxiety behaviors are likely influenced by additional neural and hormonal pathways as well as various molecular mechanisms. For instance, oncogenic signaling pathways such as the PI3K/AKT-Wnt/β-catenin pathway in the liver, and the role of Lgr5, may be significant contributors to the pathogenesis of anxiety disorders [62]. These pathways are regulated by glucocorticoids, suggesting that the activity of 11β-HSD-1 could be closely linked with these molecular pathways [63,64].

RES, despite its well-documented therapeutic benefits, is not metabolically stable, which has hindered its development as an effective clinical drug. The limited bioavailability and extensive metabolism of drugs that show efficacy in vitro remain significant challenges in translating these promising compounds into clinical trials. Current strategies to enhance the bioavailability and stability of resveratrol include chemical derivatization, encapsulation in nanoparticles or liposomes, and combination therapy with other drugs [65]. There have been a few reports on the combination of resveratrol with selective serotonin reuptake inhibitors for the treatment of stress-related anxiety [66]. Research in our laboratories has recently begun to explore these combination therapies, and this will be the focus of our future studies.

This study was primarily conducted in a rat model of stress-related anxiety. Unfortunately, the physiological and psychological differences between animal models and humans suggest that these results may not directly translate to clinical treatment strategies. However, studies in humans have reported that 11β-HSD-1 and MAO are significant markers of anxiety-like symptoms in PTSD and stress-related anxiety [67,68]. These findings highlight the relevance of targeting these markers in human research and suggest that this approach may be applicable to clinical treatment.

Overall, this study suggests the potential of using RES to correct anxiety-like behavior. Due to its many protective effects, RES is as effective as antidepressants, neuroleptics, sedatives, and other drugs used for this purpose. However, the broader use of RES in treating behavioral disorders has been limited by its high metabolic rate, leading to rapid inactivation. Consequently, significant effects of RES have primarily been observed at high doses. In this study, we employed the maximum dose of RES. Even under these conditions, the concentration of the RES metabolite was an order of magnitude higher than that of the drug itself. The development of more stable forms of RES is currently underway, and we anticipate that these formulations will have significant medical applications in the future.

Ultimately, liver 11β-HSD-1 and brain MAO are two critical systems in the metabolic regulation of the LHPA axis. Studying these systems not only enhances our understanding of the biochemical foundations of stress-related anxiety but also reveals new opportunities for developing therapeutic interventions that could be more effective in treating anxiety disorders. This integrative approach underscores the potential of RES as a multitarget treatment, providing a promising alternative to traditional therapies.

## 5. Conclusions

Resveratrol has unique capabilities in addressing psychiatric, neurological, and neurodegenerative diseases. Beyond the enzymes discussed here, RES can target a broad range of molecular pathways. We hypothesize that these unique properties are due to its ability to modulate gene networks, which involve coordinated activity among groups of genes. In PTSD and stress-related anxiety, disruption of these gene networks is central to pathogenesis. To further investigate these hypotheses, we plan to employ bioinformatics, genomics, transcriptomics, and metabolomics approaches. Consequently, our upcoming research on the mechanisms underlying RES’s protective effects will have an inherently interdisciplinary character, potentially expanding the use of RES as a therapeutic agent.

The current study validates the antianxiogenic effects of resveratrol in a chronic predator stress paradigm. These effects are mediated through RES targeting 11β-HSD-1 in the liver and MAO-A/MAO-B in the brain, as supported by the negative correlations observed between plasma RES concentration and the activities of these enzymes. PS serves as a trigger for post-traumatic stress disorder, and recent research has demonstrated that RES alleviates PTSD-like anxiety induced by prolonged exposure to predator odor. This effect is likely due to RES’s action on these enzymes.

In silico data suggest that RES binds with high affinity to specific sites on MAO enzymes and 11β-HSD-1, further supporting its role in modulating these targets. However, while in silico findings are valuable, the current results highlight RES’s capacity to influence essential biological processes such as gene expression and mitochondrial function. The observed antianxiogenic effects of RES treatment are thus closely linked to its hepatoprotective properties.

Future research will focus on elucidating the relationship between RES treatment and its impact on MAO and 11β-HSD-1 activity, including its interaction with endogenous mediators such as SIRT1, AMPK, BDNF, PGC-1α, NHLH2, and relevant microRNAs. These studies will further clarify the mechanisms underlying RES’s therapeutic effects.

The results suggest that RES may have broader applications beyond PTSD-associated anxiety, potentially extending its therapeutic benefits to other anxiety disorders. This could broaden its utility in psychiatric treatment.

## Figures and Tables

**Table 1:** Impact of resveratrol treatment on the behavior of PS-subjected rats.

	Control (*n* = 8)	PS ^a^ (*n* = 7)	PS + RES ^b^ (*n* = 7)
Time spent in open arms (s)	193.37 ± 41.44	83.42 ± 27.64 **	176.71 ± 48.35 ^##^
Time spent in closed arms (s)	106.62 ± 41.44	216.57 ± 27.6 **	137.57 ± 48.35 ^##^
Entries in open arms	1.87 ± 0.96	1.71 ± 1.01	3.14 ± 0.68
Entries in closed arms	2.0 ± 0.89	5.14 ± 1.51 **	3.14 ± 0.35 ^##^
AI ^c^	0.38 ± 0.09	0.76 ± 0.8 **	0.45 ± 0.15 ^##^
Freezing	0.36 ± 0.09	2.42 ± 0.3 ***	0 ± 0 ^###^

^a^ = predator stress; ^b^ = predator stress + resveratrol; ^c^ = anxiety index; * = effect between groups PS and Control; ^#^ = effect between groups PS and PS + RES; ** *p* < 0.01; *** *p* < 0.001; ^##^ *p* < 0.01; ^###^ *p* < 0.001.

**Table 2:** Impact of predator stress and resveratrol treatment on liver injury performance. Data are given as mean ± standard deviation.

Group	Necrosis ^a^	Macrophages ^a^	Lymphocytes ^a^
Control	0 ± 0	1.05 ± 0.15	0.7 ± 0
PS	2.98 ± 0.43 ***	2.25 ± 0.05 **	3.1 ± 0.29 ^**^
PS + RES	0.3 ± 0.07 ^###^	0.49 ± 0.11 ^###^	0.88 ± 0.11

^a^ = pathology score; * = effect between groups PS and control; ^#^ = effect between groups PS and RES; ** *p* < 0.01; *** *p* < 0.001; ^###^ *p* < 0.001.

## Data Availability

The original contributions presented in the study are included in the article; further inquiries can be directed to the corresponding authors.

## References

[1] Saviola F., Pappaianni E., Monti A., Grecucci A., Jovicich J., De Pisapia N. (2020). Trait and state anxiety are mapped differently in the human brain. Sci. Rep..

[2] Chu B., Marwaha K., Sanvictores T., Awosika A.O., Ayers D. (2024). Physiology, Stress Reaction.

[3] Ramsay D.S., Woods S.C. (2014). Clarifying the roles of homeostasis and allostasis in physiological regulation. Psychol. Rev..

[4] Korte S.M., Koolhaas J.M., Wingfield J.C., McEwen B.S. (2005). The Darwinian concept of stress: Benefits of allostasis and costs of allostatic load and the trade-offs in health and disease. Neurosci. Biobehav. Rev..

[5] Krupnik V. (2021). Depression as a failed anxiety: The continuum of precision-weighting dysregulation in affective disorders. Front. Psychol..

[6] Tseilikman V., Komelkova M., Lapshin M., Alliluev A., Tseilikman O., Karpenko M., Pestereva N., Manukhina E., Downey H.F., Kondashevskaya M. (2020). High and low anxiety phenotypes in a rat model of complex post-traumatic stress disorder are associated with different alterations in regional brain monoamine neurotransmission. Psychoneuroendocrinology.

[7] Farach F.J., Pruitt L.D., Jun J.J., Jerud A.B., Zoellner L.A., Roy-Byrne P.P. (2012). Pharmacological treatment of anxiety disorders: Current treatments and future directions. J. Anxiety Disord..

[8] Wilson C.B., McLaughlin L.D., Ebenezer P.J., Nair A.R., Dange R., Harre J.G., Shaak T.L., Diamond D.M., Francis J. (2014). Differential effects of sertraline in a predator exposure animal model of post-traumatic stress disorder. Front. Behav. Neurosci..

[9] Yuan Y., Zhen L., Li Z., Xu W., Leng H., Xu W., Zheng V., Luria V., Pan J., Tao Y. (2020). *trans*-Resveratrol ameliorates anxiety-like behaviors and neuropathic pain in mouse model of post-traumatic stress disorder. J. Psychopharmacol..

[10] Gambini J., Inglés M., Olaso G., Lopez-Grueso R., Bonet-Costa V., Gimeno-Mallench L., Mas-Bargues C., Abdelaziz K.M., Gomez-Cabrera M.C., Vina J. (2015). Properties of resveratrol:In vitro and in vivo studies about metabolism, bioavailability, and biological effects in animal models and humans. Oxidative Med. Cell. Longev..

[11] Shayganfard M. (2020). Molecular and biological functions of resveratrol in psychiatric disorders: A review of recent evidence. Cell Biosci..

[12] Prakash V., Bose C., Sunilkumar D., Cherian R.M., Thomas S.S., Nair B.G. (2024). Resveratrol as a promising nutraceutical: Implications in gut microbiota modulation, inflammatory disorders, and colorectal cancer. Int. J. Mol. Sci..

[13] Cao D., Wang M., Qiu X., Liu D., Jiang H., Yang N., Xu R.M. (2015). Structural basis for allosteric, substrate-dependent stimulation of SIRT1 activity by resveratrol. Genes Dev..

[14] Cantó C., Auwerx J. (2009). PGC-1*α*, SIRT1 and AMPK, an energy sensing network that controls energy expenditure. Curr. Opin. Lipidol..

[15] Herzig S., Shaw R.J. (2017). AMPK: Guardian of metabolism and mitochondrial homeostasis. Nat. Rev. Mol. Cell Biol..

[16] Price N., Gomes A., Ling A., Duarte F., Martin-Montalvo A., North B., Agarwal B., Ye L., Ramadori G., Teodoro J. (2012). SIRT1 is required for AMPK activation and the beneficial effects of resveratrol on mitochondrial function. Cell Metab..

[17] Zhang F., Lu Y.F., Wu Q., Liu J., Shi J.S. (2012). Resveratrol promotes neurotrophic factor release from astroglia. Exp. Biol. Med..

[18] Huang H., Wang Z.J., Zhang H.B., Liang J.X., Cao W.D., Wu Q., He C.P., Chen C. (2019). The function of PPAR*γ*/AMPK/SIRT-1 pathway in inflammatory response of human articular chondrocytes stimulated by advanced glycation end products. Biol. Pharm. Bull..

[19] Wang J., Zhao P., Cheng P., Zhang Z., Yang S., Wang J., Wang X., Zhu G. (2024). Exploring the effect of Anshen Dingzhi prescription on hippocampal mitochondrial signals in single prolonged stress mouse model. J. Ethnopharmacol..

[20] Novak J., Tseilikman V.E., Tseilikman O.B., Lazuko S.S., Belyeva L.E., Rahmani A., Fedotova J. (2023). Can resveratrol influence the activity of 11*β*-hydroxysteroid dehydrogenase type 1? A combined in silico and in vivo study. Pharmaceuticals.

[21] Wheelan N., Seckl J.R., Yau J.L. (2022). 11*β*-Hydroxysteroid dehydrogenase 1 deficiency prevents PTSD-like memory in young adult mice. Psychoneuroendocrinology.

[22] Tseilikman V., Dremencov E., Tseilikman O., Pavlovicova M., Lacinova L., Jezova D. (2019). Role of glucocorticoid- and monoamine-metabolizing enzymes in stress-related psychopathological processes. Stress.

[23] Williamson J.B., Jaffee M.S., Jorge R.E. (2021). Posttraumatic stress disorder and anxiety-related conditions. Contin. Lifelong Learn. Neurol..

[24] McEwen B.S., Eiland L., Hunter R.G., Miller M.M. (2012). Stress and anxiety: Structural plasticity and epigenetic regulation as a consequence of stress. Neuropharmacology.

[25] Resick P.A., Bovin M.J., Calloway A.L., Dick A.M., King M.W., Mitchell K.S., Suvak M.K., Wells S.Y., Stirman S.W., Wolf E.J. (2012). A critical evaluation of the complex PTSD literature: Implications for DSM-5. J. Trauma. Stress.

[26] Tseilikman V.E., Fedotova J.O., Tseilikman O.B., Novak J., Karpenko M.N., Maistrenko V.A., Lazuko S.S., Belyeva L.E., Kamel M., Buhler A.V. (2023). Resistance to resveratrol treatment in experimental PTSD is associated with abnormalities in hepatic metabolism of glucocorticoids. Int. J. Mol. Sci..

[27] Tseilikman V., Lapshin M., Klebanov I., Chrousos G., Vasilieva M., Pashkov A., Fedotova J., Tseilikman D., Shatilov V., Manukhina E. (2022). The link between activities of hepatic 11*β*-hydroxysteroid dehydrogenase-1 and monoamine oxidase-A in the brain following repeated predator stress: Focus on heightened anxiety. Int. J. Mol. Sci..

[28] Lazuko S.S., Kuzhel O.P., Belyaeva L.E., Manukhina E.B., Fred Downey H., Tseilikman O.B., Komelkova M.V., Tseilikman V.E. (2017). Posttraumatic stress disorder disturbs coronary tone and its regulatory mechanisms. Cell. Mol. Neurobiol..

[29] Tseilikman V.E., Kozochkin D.A., Sinitskii A.I., Tseylikman O.B., Lapshin M.S., Kuzina O.V., Komel’kova M.V., Telesheva I.B. (2016). Effect of repeated 1-h episodes of immobilization stress on activity of glucocorticoid metabolism enzymes in the liver. Bull. Exp. Biol. Med..

[30] Satav J.G., Katyare S.S. (1982). Effect of experimental thyrotoxicosis on oxidative phosphorylation in rat liver, kidney and brain mitochondria. Mol. Cell. Endocrinol..

[31] Tipton K.F., Davey G., Motherway M. (2000). Monoamine oxidase assays. Curr. Protoc. Pharmacol..

[32] Volchegorskii I.A., Rassokhina L.M., Miroshnichenko I.Y. (2013). Dynamics of lipid peroxidation—Antioxidant defense system during alloxan diabetes in rats. Bull. Exp. Biol. Med..

[33] Korourian S., Hakkak R., Ronis M.J., Shelnutt S.R., Waldron J., Ingelman-Sundberg M., Badger T.M. (1999). Diet and risk of ethanol-induced hepatotoxicity: Carbohydrate-fat relationships in rats. Toxicol. Sci..

[34] Tseilikman V.E., Shatilov V.A., Zhukov M.S., Buksha I.A., Epitashvily A.E., Lipatov I.A., Aristov M.R., Koshelev A.G., Karpenko M.N., Traktirov D.S. (2023). Limited cheese intake paradigm replaces patterns of behavioral disorders in experimental PTSD: Focus on resveratrol supplementation. Int. J. Mol. Sci..

[35] Shih J.C., Chen K., Ridd M.J. (1999). Monoamine oxidase: From genes to behavior. Annu. Rev. Neurosci..

[36] Chen K., Holschneider D.P., Wu W., Rebrin I., Shih J.C. (2004). A spontaneous point mutation produces monoamine oxidase A/B knock-out mice with greatly elevated monoamines and anxiety-like behavior. J. Biol. Chem..

[37] Glover V. (1998). Function of endogenous monoamine oxidase inhibitors (tribulin). MAO—The Mother of All Amine Oxidases.

[38] Tseilikman O.B., Kozochkin D.A., Manukhina E.B., Downey H.F., Misharina M.E., Komelkova M.V., Nikitina A.A., Golodnii S.V., Dodohova M.A., Tseilikman V.E. (2016). Predicting anxiety responses to halogenated glucocorticoid drugs using the hexobarbital sleep time test. Stress.

[39] Lee B., Choi G.M., Sur B. (2020). Antidepressant-like effects of hesperidin in animal model of post-traumatic stress disorder. Chin. J. Integr. Med..

[40] Higuchi Y., Soga T., Parhar I.S. (2018). Potential roles of microRNAs in the regulation of monoamine oxidase A in the brain. Front. Mol. Neurosci..

[41] Libert S., Pointer K., Bell E., Das A., Cohen D., Asara J., Kapur K., Bergmann S., Preisig M., Otowa T. (2011). SIRT1 activates MAO-A in the brain to mediate anxiety and exploratory drive. Cell.

[42] Ooi J., Hayden M.R., Pouladi M.A. (2014). Inhibition of excessive monoamine oxidase A/B activity protects against stress-induced neuronal death in Huntington disease. Mol. Neurobiol..

[43] Pizzinat N., Copin N., Vindis C., Parini A., Cambon C. (1999). Reactive oxygen species production by monoamine oxidases in intact cells. Naunyn-Schmiedeberg Arch. Pharmacol..

[44] Kaludercic N., Carpi A., Nagayama T., Sivakumaran V., Zhu G., Lai E.W., Bedja D., De Mario A., Chen K., Gabrielson K.L. (2014). Monoamine oxidase B prompts mitochondrial and cardiac dysfunction in pressure overloaded hearts. Antioxidants Redox Signal..

[45] Medvedev A.E., Rajgorodskaya D.I., Gorkin V.Z., Fedotova I.B., Semiokhina A.F. (1992). The role of lipid peroxidation in the possible involvement of membrane-Bound monoamine oxidases in gamma-aminobutyric acid and glucosamine deamination in rat brain: Focus on chemical pathogenesis of experimental audiogenic epilepsy. Mol. Chem. Neuropathol..

[46] Dean R.T., Thomas S.M., Garner A. (1986). Free-radical-mediated fragmentation of monoamine oxidase in the mitochondrial membrane Roles for lipid radicals. Biochem. J..

[47] Komelkova M., Manukhina E., Downey H.F., Sarapultsev A., Cherkasova O., Kotomtsev V., Platkovskiy P., Fedorov S., Sarapultsev P., Tseilikman O. (2020). Hexobarbital sleep test for predicting the susceptibility or resistance to experimental posttraumatic stress disorder. Int. J. Mol. Sci..

[48] Tseilikman V., Dremencov E., Maslennikova E., Ishmatova A., Manukhina E., Downey H.F., Klebanov I., Tseilikman O., Komelkova M., Lapshin M.S. (2019). Post-traumatic stress disorder chronification via monoaminooxidase and cortisol metabolism. Horm. Metab. Res..

[49] Liu D., Xie K., Yang X., Gu J., Ge L., Wang X., Wang Z. (2014). Resveratrol reverses the effects of chronic unpredictable mild stress on behavior, serum corticosterone levels and BDNF expression in rats. Behav. Brain Res..

[50] Rasouri S., Lagouge M., Auwerx J. (2007). SIRT1/PGC-1: Un axe neuroprotecteur?. Médecine/Sci..

[51] Yu D., Zhao X.Y., Meng Q.P., Teng D., Deng K., Lin N. (2022). Resveratrol activates the SIRT1/PGC-1 pathway in mice to improve synaptic-related cognitive impairment after TBI. Brain Res..

[52] Lagouge M., Argmann C., Gerhart-Hines Z., Meziane H., Lerin C., Daussin F., Messadeq N., Milne J., Lambert P., Elliott P. (2006). Resveratrol improves mitochondrial function and protects against metabolic disease by activating SIRT1 and PGC-1*α*. Cell.

[53] Li W., Guo B., Tao K., Li F., Liu Z., Yao H., Feng D., Liu X. (2019). Inhibition of SIRT1 in hippocampal CA1 ameliorates PTSD-like behaviors in mice by protections of neuronal plasticity and serotonin homeostasis via NHLH2/MAO-A pathway. Biochem. Biophys. Res. Commun..

[54] El-Hawary S.S., Sayed A.M., Issa M.Y., Ebrahim H.S., Alaaeldin R., Elrehany M.A., Abd El-Kadder E.M., Abdelmohsen U.R. (2021). Anti-Alzheimer chemical constituents of Morus macroura Miq.: Chemical profiling, in silico and in vitro investigations. Food Funct..

[55] Yáñez M., Fraiz N., Cano E., Orallo F. (2006). Inhibitory effects of cis- and trans-resveratrol on noradrenaline and 5-hydroxytryptamine uptake and on monoamine oxidase activity. Biochem. Biophys. Res. Commun..

[56] Basheer L., Schultz K., Guttman Y., Kerem Z. (2017). In silico and in vitro inhibition of cytochrome P450 3A by synthetic stilbenoids. Food Chem..

[57] Tseilikman V., Kozochkin D., Synitsky A., Sibiriak S., Tseilikman O., Katashinsky E., Nikitina A., Vinogradov D., Simbirtsev A. (2012). Does stress-induced release of interleukin-1 cause liver injury?. Cell. Mol. Neurobiol..

[58] Xie H., Xie D., Zhang J., Jin W., Li Y., Yao J., Pan Z., Xie D. (2020). ROS/NF-*κ*B signaling pathwaymMediated transcriptional activation of TRIM37 promotes HBV-associated hepatic fibrosis. Mol. Ther. Nucleic Acids.

[59] Chen Y., Li J., Zhang M., Yang W., Qin W., Zheng Q., Chu Y., Wu Y., Wu D., Yuan X. (2022). 11*β*-HSD1 inhibitor alleviates non-alcoholic fatty liver disease by activating the AMPK/SIRT1 signaling pathway. Nutrients.

[60] Pshenichnyuk S.A., Komolov A.S. (2015). Dissociative electron attachment to resveratrol as a likely pathway for generation of the H2 antioxidant species inside mitochondria. J. Phys. Chem. Lett..

[61] Zeqiraj E., Filippi B.M., Deak M., Alessi D.R., van Aalten D.M.F. (2009). Structure of the LKB1-STRAD-MO25 complex reveals an allosteric mechanism of kinase activation. Science.

[62] He J., Han J., Lin K., Wang J., Li G., Li X., Gao Y. (2023). PTEN/AKT and Wnt/*β*-catenin signaling pathways regulate the proliferation of Lgr5+ cells in liver cancer. Biochem. Biophys. Res. Commun..

[63] Snyder J.C., Pack T.F., Rochelle L.K., Chakraborty S.K., Zhang M., Eaton A.W., Bai Y., Ernst L.A., Barak L.S., Waggoner A.S. (2015). A rapid and affordable screening platform for membrane protein trafficking. BMC Biol..

[64] Hu J., Mao Z., He S., Zhan Y., Ning R., Liu W., Yan B., Yang J. (2017). Icariin protects against glucocorticoid induced osteoporosis, increases the expression of the bone enhancer DEC1 and modulates the PI3K/Akt/GSK3*β*/*β*-catenin integrated signaling pathway. Biochem. Pharmacol..

[65] Salla M., Karaki N., El Kaderi B., Ayoub A.J., Younes S., Abou Chahla M.N., Baksh S., El Khatib S. (2024). Enhancing the bioavailability of resveratrol: Combine it, derivatize it, or encapsulate it?. Pharmaceutics.

[66] Sakr H.F., Abbas A.M., Elsamanoudy A.Z., Ghoneim F.M. (2015). Effect of fluoxetine and resveratrol on testicular functions and oxidative stress in a rat model of chronic mild stress-induced depression. J. Physiol. Pharmacol..

[67] Pivac N., Knezevic J., Kozaric-Kovacic D., Dezeljin M., Mustapic M., Rak D., Matijevic T., Pavelic J., Muck-Seler D. (2007). Monoamine oxidase (MAO) intron 13 polymorphism and platelet MAO-B activity in combat-related posttraumatic stress disorder. J. Affect. Disord..

[68] Yehuda R., Seckl J. (2011). Minireview: Stress-related psychiatric disorders with low cortisol levels: A metabolic hypothesis. Endocrinology.

